# Sprouts and Needles of Norway Spruce (*Picea abies* (L.) Karst.) as Nordic Specialty—Consumer Acceptance, Stability of Nutrients, and Bioactivities during Storage

**DOI:** 10.3390/molecules25184187

**Published:** 2020-09-12

**Authors:** Tuula Jyske, Eila Järvenpää, Susan Kunnas, Tytti Sarjala, Jan-Erik Raitanen, Maarit Mäki, Helena Pastell, Risto Korpinen, Janne Kaseva, Tuomo Tupasela

**Affiliations:** 1 Natural Resources Institute Finland (Luke), Tietotie 2, FI-02150 Espoo, Finland; susan.kunnas@luke.fi (S.K.); jan-erik.raitanen@helsinki.fi (J.-E.R.); risto.korpinen@luke.fi (R.K.); 2 Natural Resources Institute Finland (Luke), Myllytie 1, FI-31600 Jokioinen, Finland; eila.jarvenpaa@luke.fi (E.J.); maarit.maki@luke.fi (M.M.); janne.kaseva@luke.fi (J.K.); tuomo.tupasela@luke.fi (T.T.); 3 Natural Resources Institute Finland (Luke), Kaironiementie 15, FI-39700 Parkano, Finland; tytti.sarjala@luke.fi; 4 Department of Chemistry, University of Helsinki, P.O. Box 55, FI-00014 Helsinki, Finland; 5 Finnish Food Authority, Mustialankatu 3, FI-00790 Helsinki, Finland; helena.pastell@foodauthority.fi

**Keywords:** antioxidative, color, drying, flavor, microbial quality, nutritive value, safety, vitamins

## Abstract

Developing shoots, i.e., sprouts, and older needles of Norway spruce (*Picea abies* (L.) Karst.) have traditionally been used for medicinal purposes due to the high content of vitamins and antioxidants. Currently, sprouts are available as, for example, superfood and supplements. However, end-product quality and nutritive value may decline in the value-chain from raw material sourcing to processing and storage. We studied (1) impacts of different drying and extraction methods on nutritional composition and antioxidative properties of sprouts and needles, (2) differences between sprouts and needles in nutritional composition and microbiological quality, and (3) production scale quality of the sprouts. Additionally, (4) sprout powder was applied in products (ice-cream and sorbet) and consumer acceptance was evaluated. According to our results, older needles have higher content of dry matter, energy, and calcium, but lower microbial quality than sprouts. Sprouts showed a higher concentration of vitamin C, magnesium, potassium, and phosphorus than older needles. Freeze-drying was the best drying method preserving the quality of both sprouts and needles, e.g., vitamin C content. The antioxidative activity of the sprout extracts were lower than that of needles. Ethanol-water extraction resulted in a higher content of active compounds in the extract than water extraction. Sensory evaluation of food products revealed that on average, 76% of consumers considered sprout-containing products very good or good, and a creamy product was preferred over a water-based sorbet.

## 1. Introduction

Traditionally, sprouts, i.e., new developing shoots of Norway spruce (*Picea abies* (L.) Karst.), have been eaten as such, used as herbal tea or in folk medicine in different ways, e.g., to prevent respiratory diseases and stomach ailments in Finland and central Europe [1,2,3]. The sprouts are especially rich in vitamin C and minerals [2,4,5]. The sprouts also have high contents of secondary metabolites such as flavonoids (e.g., kaempferol, quercetin, isorhamnetin, and myricetin), condensed tannins, stilbenes, and terpenoids [2,5,6,7,8,9]. Due to the richness of vitamin C and phenolics, sprouts and needles have shown antioxidative activity [9,10,11]. Dietary intake of the compounds of sprouts may thus have multiple health benefits [2,12]. The research topic is timely now, as sprouts are gaining popularity among wild herbs used as a Nordic piquancy ingredient in different culinary products, such as snacks, jams, syrups, alcoholic and non-alcoholic beverages, or in superfood and supplements, and as essential oils with antimicrobial properties [3,13]. In recent years, Finnish restaurants have also expressed an increased interest in utilizing the sprouts as ingredients for a variety of uses due to their unique taste [14,15,16,17].

The Finnish forests provide a great potential to increase the harvest of pure, high-quality sprout raw material—without impurities or pollutants—for the development of added-value products. This is because, in Finland, a large portion (e.g., 99.9% in Northern Finland) of the forest area meets the requirements for organic certification and 9 million ha of forest in Lapland is already certified as organic wild collection area [18,19]. Value-chain development for sprouts would also provide forest owners with a new potential source of income, especially if growth in export markets is to be expected. It has also been shown that due to the harvesting of sprouts, only little adverse effect on spruce growth and subsequent felling revenue is to be expected, especially if the end shoots at the top of the tree are left uncollected and harvesting is carried out only in one year [20]. However, challenges and knowledge gaps still exist, hindering the development of the value-chains from harvesting to industrial processing and storage. 

First, the annual time for sprout harvesting is very short. The development of new branches of Norway spruce in Finland begins with the development and growth of primordial shoots in buds, followed by bud burst in mid-May to early-June [7,21]. The timing is dependent on the geographical location of the growing site, while the environmental predeterminants are mainly photoperiod and temperature sum accumulation [21,22,23,24,25,26]. The intensive growth period of the light-green, new shoots thus only lasts for a few weeks, and the shoots typically reach their final length by mid-summer [7,17]. After that, the color of the prolonged needles darkens, they harden, and the taste becomes more bitter [7,17]. Thus, the intensive growing period is the only annual time for the manual harvesting of the sprouts, being typically only two to three weeks per year. As the harvest requires manual labor and a permission from the landowner is needed (i.e., there is no every man’s right to collect sprouts), there is no well-established supply chain of sprouts in Finland [17]. This causes a challenge for the companies and industry willing to utilize larger amounts of sprouts as raw material [15,17]. The average harvest amount is one ton; however, progressive small- and middle-sized enterprises (SMEs) harvest sprouts at 3000–5000 kg during the harvest season. Furthermore, restaurants seem to prefer smaller sprouts (15–30 mm) over larger ones (30–60 mm). This obviously leads to a less-efficient harvesting, i.e., more trees are needed with larger harvested forest area to obtain the same sprout mass than when collecting larger sprouts [17]. One possible solution for the challenge would be better raw material sorting for different end uses and/or development of techniques that allow utilization of larger sprouts or even mature needles for certain end-products.

Secondly, there is a lack of knowledge on treatment techniques, i.e., drying being a crucial phase in the production chain to preserve the raw materials and support creating quality products. As the annual sprout harvest is collected within a very short period of time, the raw material must be properly conserved and stored to maintain chemical and microbial quality, as well as nutritive value, color, taste and structure during storage, further processing, and also end-products’ desired shelf-lives. SMEs often lack knowledge on the best processing practices, i.e., effects of drying parameters on raw material quality, or they do not have the required technology and infrastructure. For example, when the SMEs output of the freeze-dried spruce powder is a few hundreds of kilograms, it is more profitable to outsource parts of the spruce processing. That involves freezing, transportation, and freeze-drying. In Finland, the freeze-drying is commonly offshored. Therefore, the quality controlling becomes more difficult but also more important. When something goes wrong during one stage of processing, the damage can be irreversible.

Thirdly, there is a lack of research-based knowledge on the chemical properties and nutritive value of sprouts, but especially those of needles. Sprouts and needles have often been treated as a homogeneous biomass and characterized together. Ample literature exists reporting (eco)physiological aspects of nonedible spruce needles, whereas only a limited number of studies analyzing their properties for food industry needs have been published [8,10].

This study explored ways to overcome these above-mentioned challenges caused by a short harvesting period of the spruce sprouts. Thus, we studied:

1. What is the optimal way to dry sprout raw material to preserve the quality (flavor, color, antioxidative activity, and vitamins)?
2. Can the sprouts be replaced by older, i.e., mature, needles, and how do the quality and basic characteristics differ between sprouts and older needles (from herein called ‘needles’)?
3. How do the quality factors change during the large trader process and what needs to be considered from a quality point of view?
4. How are the sprout-containing gourmet food products (ice-cream and sorbet) accepted by the consumers?

## 2. Results and Discussion

### 2.1. Drying Results

The dry matter content was determined from fresh and differently dried samples of Norway spruce sprouts and older needles. The results are shown in Table 1. 

### 2.2. Basic Properties of Spruce Sprouts and Needles 

#### 2.2.1. Macro-Components and Carbohydrates

**Figure 1:**
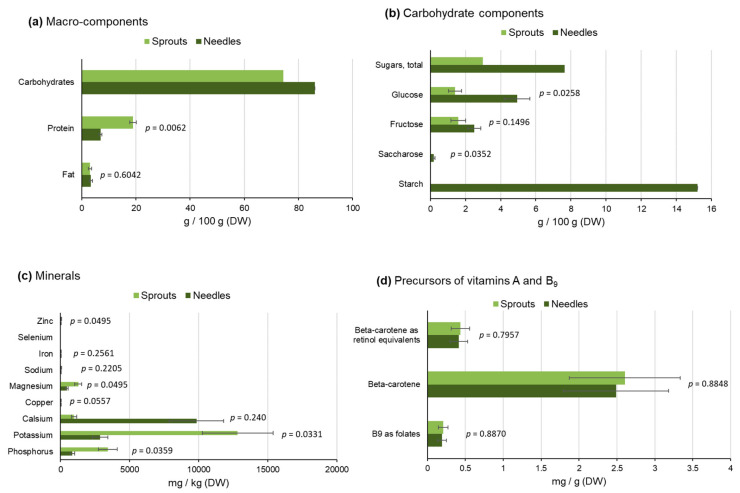

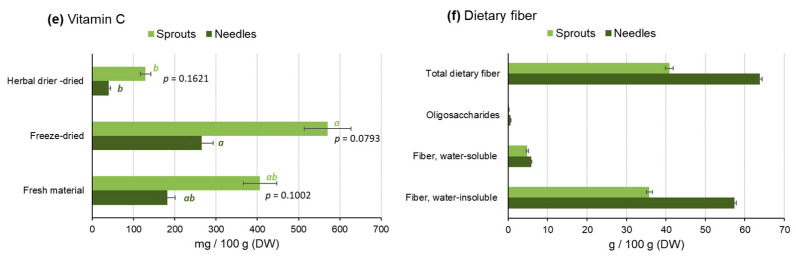
Quantitative analysis results of basic properties of sprouts and needles of Norway spruce: macro-components (**a**), carbohydrate composition (**b**), minerals (**c**), vitamin A and B_9_ precursors (**d**), vitamin C (**e**), and dietary fiber (**f**). Note: vitamin C content is shown for differently treated raw materials (**e**). The error bars indicate the standard deviations. The *p*-values for statistical difference between sprouts and needles are presented. Different letters in (**e**) refer to statitistically signficiant (*p* < 0.05) differences in vitamin C content of sprouts (light green) and needles (dark green) between different drying methods.

The freshly collected sprouts of Norway spruce had 2.7 times higher content of proteins (18.9 g/100 g dry weight (DW) vs. 7 g/100 g DW) than that in older needles. In contrast, the amount of carbohydrates was 1.2 times higher in the needles than in the sprouts, while fat content was ca. 3% in both raw materials (Figure 1a). The carbohydrate composition significantly differed between the studied raw materials: the needles had 2.6 times higher total amount of soluble sugars (SS) and 8 times higher starch content than that in the sprouts (Figure 1b). Glucose and fructose were the major monosaccharides in both raw materials but showed notably higher concentrations in the needles than in the sprouts (4.9 g/100 g DW vs. 1.4 g/100 g DW, and 2.5 g/100 g DW vs. 1.6 g/100 g DW, respectively).

The content of total non-structural carbohydrates (i.e., soluble sugars (SS) and starch) of conifer needles shows a strong seasonal pattern, especially in the boreal vegetation zone [27]. Starch is the storage compound of conifer needles that typically peaks around late spring–early summer. In contrast, SS show the highest amounts in winter and early spring (to protect the tissues from freezing), and the lowest values during late spring–mid-summer during the active period of annual growth [27]. The concentrations of needle starch and SS observed in our study are within the range of values reported in the literature for boreal and temperate conifers during the same phenological phase of the annual cycle [28,29].

#### 2.2.2. Minerals and Vitamins

The content of ash-forming compounds was especially high in the sprouts of spruce: the highest concentrations were detected for potassium, phosphorus, and magnesium (Figure 1c). In the needles, the calcium concentration exceeded that found in the sprouts (Figure 1c). Similar differences in mineral contents between the sprouts and needles of Norway spruce have also been detected by Werkelin et al. [4]. 

Folates (vitamin B_9_) and beta-carotene (vitamin A precursor) were detected as minor compounds in both sprouts and older needles of spruce (Figure 1d). In contrast, both raw materials were rich in ascorbic acid (vitamin C) (Figure 1e). 

The vitamin C values for fresh sprouts and needles were 406.7 mg/100 g DW and 182.5 mg/100 g DW, respectively. Freeze-dried material contained approximately 30% more vitamin C as compared to fresh material, while freeze-drying resulted in 4- and 7-fold vitamin C values of sprouts and needles as compared to herbal dryer-dried raw materials (Figure 1e). This indicates that appropriately conducted freeze-drying process is the optimal way for preserving the vitamin C for storage. 

The measured vitamin C values fit into the range of those reported for developing coniferous needles, although the measured vitamin C content of freeze-dried sprouts showed at the higher end of the range of vitamin C content. Radulescu et al. [5] followed the seasonal accumulation of ascorbic acid in the young shoots in different tree species (samplings in mid-May, mid-July, and early October), and found that the highest vitamin C contents were mainly found from the samples collected in autumn: 301.5 mg/100 g DW in *Picea abies*, 275.1 mg/100 g DW in *Pseudotsuga menziesii*, 231.3 mg/100 g DW in *Pinus nigra*, and 158.8 mg/100 g DW in *Abies alba*.

#### 2.2.3. Dietary Fiber

The total dietary fiber (DF) content was high in both spruce sprouts and needles, 40.9 and 63.8 g/100 g (dry weight), respectively (Figure 1f). Expressed in fresh weights, the difference in DF content between sprouts and needles was much larger, 7.2 and 36.5 g/100 g respectively, because sprouts contain twice the amount of water compared to needles. Nearly 88% of total DF in sprouts and 90% in needles is composed of water-insoluble polysaccharides, such as cellulose and linear hemicelluloses. Also, some water-soluble polysaccharides, e.g., highly substituted hemicelluloses, were found in spruce sprouts (4.9 g/100 g DW) and needles (5.9 g/100 g DW). Oligosaccharides were detected at only 0.2 g/100 g DW in sprouts and 0.6 g/100 g DW in needles.

There is only very limited information in the scientific literature about the fiber content of spruce needles, and this is the first report to present their dietary fiber composition in detail. The results found are not fully comparable with the analytical results obtained now, since only some of the different dietary/crude fiber fractions have been analyzed in previous studies, and the analytical methods used in them differ significantly from those used in this study. A crude fiber content was found to be 31% (DW) in *Picea* spp. needles and 30% in needle buds (sprouts) in the study of Pendergast and Boarg [30]. The reported that crude fiber levels are significantly lower than dietary fiber contents analyzed in this study, which may be due to differences in spruce species, age of sprouts and needles, and differences in analytical methods.

### 2.3. Microbial Quality of Spruce Sprouts and Needles

According to the results, older needles had lower microbial quality than the sprouts (Table 2). As compared to sprouts, in needles, the numbers of colony forming units (cfu) were 1.8, 0.8, and 1.3 log_10_ cfu/g (fresh weight, FW) units higher for aerobic plate counts, yeasts, and molds, correspondingly. The numbers of enterobacteria were below the detection limit, <10 cfu/g, in both raw materials. The aerobic plate count of sprouts was ca. 300 cfu/g and that of the needles was 16,000 cfu/g (Table 2).

In Finland, the recommended reference values by the Finnish Food and Drink Industries’ Federation (ETL) for the maximum amount of yeasts and molds in raw edible plants and vegetables is 10,000–100,000 (cfu)/g and 1000–10,000 (cfu)/g, respectively [31]. If the lower reference value is repeatedly exceeded, the situation must be assessed by the operator. In the case the higher value is exceeded, a risk assessment must be performed, and the operator must take action [31]. According to our results of the needles, the lower reference value for the yeasts and the higher reference value for the molds was exceeded. This was probably due to the fact that the needles of Norway spruce have a naturally occurring biome of yeasts and molds, as their longevity is 5–7 years in the south and 10–14 years in Northern Finland [32,33]. The needles were collected from the tips of the lower branches, thus representing a younger part of the mature needle biomass on the tree stem.

### 2.4. Color of Fresh and Dried Spruce Sprouts and Needles

**Figure 2:**
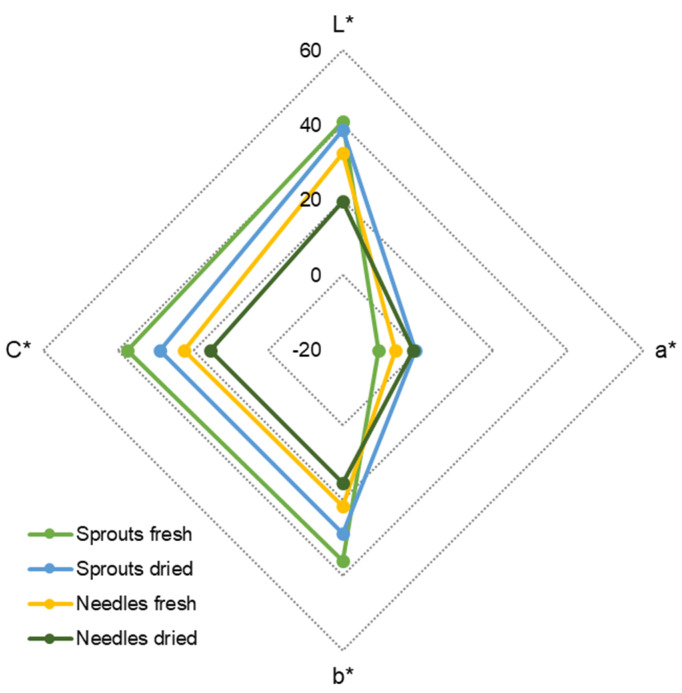
Quantitative color measurements of fresh and freeze-dried sprouts and needles of Norway spruce. L* indicates lightness, C* represents chroma, a* expresses color from green to red, and b* expresses color from blue to yellow.

The differences in colors of the raw material samples are illustrated in Figure 2. Specifically, according to the spectrophotometric color measurements (L*a*b*) and subsequent derivatives (C*), the freeze-dried sprouts and needles were darker (L*: 41 ± 5 and 38.6 ± 4 for fresh and dry sprouts, and 32.5 ± 2 and 19.7 ± 1 for fresh and dry needles), and their color intensity was lower (C*: 37.6 ± 3 and 28.9 ± 2 for fresh and dry sprouts, and 22.5 ± 1 and 15.4 ± 1 for fresh and dry needles) than that of the fresh raw materials. Difference in a* and b* was observed in both raw materials due to drying (a*: –10.6 ± 2 and –0.74 ± 1.6 for fresh and dry sprouts, and –6.06 ± 1.6 and –1.4 ± 0.54 for fresh and dry needles, respectively; b*: 36.1 ± 2.9 and 28.8 ± 2.1 for fresh and dry sprouts, and 21.6 ± 1.1 and 15.4 ± 1.1 for fresh and dry needles, respectively). 

### 2.5. Antioxidative Activity of Spruce Sprouts and Needles 

**Figure 3:**
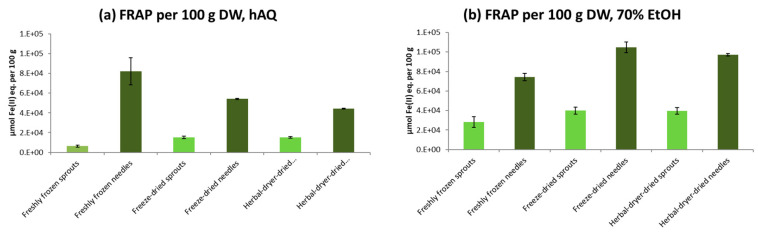
Ferric ion reducing antioxidant power (FRAP) of sprout and older needle raw materials (freshly frozen, freeze-dried, warm air-dried) of Norway spruce after water (hAQ) (**a**), and 70% ethanol (70% EtOH) (**b**) extraction. The results are calculated per 100 g dry weight (DW). The error bars indicate standard deviations.

**Figure 4:**
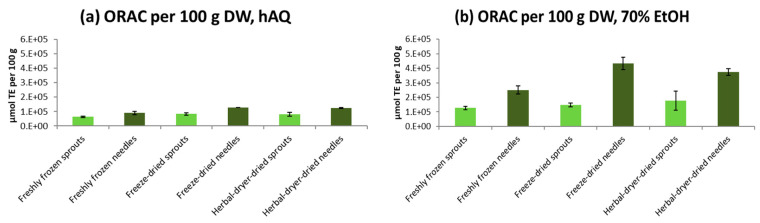
Oxygen radical absorbance capacity (ORAC) of sprout and older needle raw materials (freshly frozen, freeze-dried, warm air-dried) of Norway spruce after water (hAQ) (**a**), and 70% ethanol (70% EtOH) (**b**) extraction. The results are calculated per 100 g dry weight (DW). The error bars indicate standard deviations.

The highest antioxidative capacity was found in freeze-dried and older needles dried in an herbal dryer after ethanol extraction, as analyzed by Oxygen Radical Absorbance Capacity (ORAC) and Ferric ion Reducing Antioxidant Power (FRAP) methods (Figure 3; Figure 4). On average, 70% ethanol extraction resulted in 2.7 times higher antioxidative activity in ORAC tests and 1.9 times higher activity in FRAP tests than the extraction with water only (*p* < 0.0001 in both FRAP and ORAC). Freeze-drying was more efficient at preserving the antioxidant capacity: in comparison with the freshly frozen samples stored at –80 °C for approximately one year, freeze-dried raw materials had 1.2 times higher activity in the ORAC test and 1.6 times higher activity in the FRAP test (*p* = 0.0626 and *p* = 0.0005 for differences between drying methods in FRAP and ORAC, respectively) when the activities were calculated per dry weight. Antioxidant activity of the raw materials dried in a warm air drier showed 2.3- and 1.2-times higher activity in ORAC and FRAP tests than frozen material (as calculated per DW; *p* = 0.0006 for ORAC and *p* = 0.1104 for FRAP).

However, even when the water content was taken into account, it was shown that antioxidative properties of the sprouts were well-preserved in the drying process either in an herbal drier or by freeze-drying. Freeze-dried sprouts and needles had on average 5% and 16% higher activities in the ORAC and FRAP tests than the raw materials dried in an herbal drier, respectively (results per DW; *p* = 0.2727 for FRAP, and *p* = 0.8696 for ORAC). In the H_2_O_2_ scavenging-tests, hydrogen peroxide inhibition values were generally low (on average 8.3%), and no differences between the extraction and drying methods were observed (data not shown).

The reason for the significantly (*p* < 0.0001 in both FRAP and ORAC) higher antioxidative activities in the needles than in sprouts was probably due to the generally higher content of secondary metabolites in the needles than in the sprouts [7]. The phenolic analyses of spruce sprouts and needles remain as a topic for our next study. Besides the estimated chemical differences between the studied raw materials, the structures of mature needles (i.e., structure and chemical composition of epicuticular waxes) differ from those in developing shoots and may provide a barrier to losses of water and active compounds during storage [7,34].

The secondary metabolites of new shoots have shown to peak early during the shoot development. Condensed tannins were accumulated already in late buds, and piperidine alkaloids were found to peak in their concentration during the early phases of shoot development in Norway spruce [35,36].

Piperidine alkaloids are reported to pose toxic, teratogenic, and antifeedant properties [37,38,39,40]. These compounds hence pose a potential health risk in edible products, depending on the exposure level from the actual consumer products. On the other hand, piperidine alkaloids have also been reported to show promising antibacterial and antifungal activity [41]. More information is therefore needed on the (pre-)treatments by which different sprout compounds can be either extracted from or preserved in the raw materials for developing higher added-value, safe products for consumers.

### 2.6. Production Scale: Quality of Raw Materials Across the Value-Chain 

#### 2.6.1. Microbial Quality

The microbial or hygienic quality of the raw materials was at a good level in 2017–2019. There were no indications of *Salmonella* or *Listeria monocytogenes* bacteria, and the numbers of *Clostridium perfringens* and *Escherichia coli* bacteria were below the detection limit, <10 cfu/g, and *Bacillus cereus* bacteria were below the detection limit, <100 cfu/g. 

Furthermore, the analysis of storage life of freshly frozen spruce sprouts (–20 °C) and freeze-dried spruce sprout powder were carried out. Due to the appropriate storage during this two-year period, the microbial quality level of these batches was preserved well. There were no indications of *Salmonella* bacteria or *Listeria monocytogenes*, and the numbers of *Clostridium perfringens* and *Escherichia coli* bacteria were below the aforementioned detection limits.

#### 2.6.2. Heavy Metals and Pesticides

There were no indications of heavy metals, such as arsenic, mercury, cadmium, or lead, in the spruce sprouts during 2017–2019. According to the analysis, neither pesticides (e.g., pyrethroids and organochloride, nitrate, and phosphorus compounds) nor their traces were found; however, traces of the commercial mosquito repellent were detected in the fresh spruce sprouts (Diethyltoluamide (DEET) 0.051 ± 0.025 mg/kg and pyrethrin 0.052 ± 0.026 mg/kg) and spruce powder (DEET 0.48 ± 0.24 mg/kg and icaridin 0.072 ± 0.036) in 2019, respectively. Although the amounts of the found pesticides are near the detection limits and do not affect the safety of the foodstuff, the results highlight how extremely important the raw material quality and the early-chain processing practices are. In this case, the collectors of spruce sprouts were forbidden to use any insect repellents.

#### 2.6.3. Vitamins and Minerals

**Figure 5:**
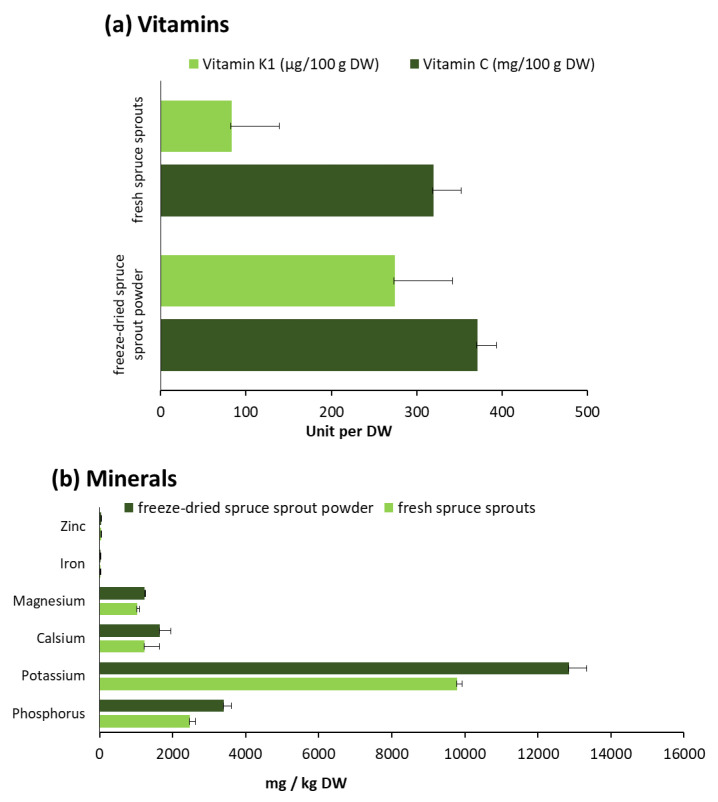
The average vitamin (**a**) and mineral (**b**) compositions of the fresh spruce sprouts and the freeze-dried spruce sprout powder in 2018–2019 (mg/kg DW). The results of the batch from 2017 are presented in Section 2.2.

The vitamin and mineral analyses (Figure 5a) show that spruce sprouts and freeze-dried spruce sprout powder are high in vitamins C and K_1_ (compared to 30% of the daily reference intake value for each 100 g of the fresh spruce sprouts (FW) and the spruce sprout powder (DW)). The daily reference intake values of vitamins and minerals are specified in Nutrient Labelling Decree 588/2009 of the Ministry of Agriculture and Forestry and in Annex XIII to Food Information Regulation 1169/2011 [42]. In addition, they are high in many minerals (Figure 5b). The results obtained during different years indicate that vitamin and mineral content of sprouts were preserved well in the freeze-drying process. In fact, there were no statistical differences in vitamin C contents or in beta carotene (vitamin A precursors total (cis + trans)) in fresh spruce sprouts and freeze-dried spruce sprout powder in years 2018 (α = 0.05, *p*-values 0.379 and 0.174, respectively) and 2019 (α = 0.05, *p*-values 0.196 and 0.141, respectively). Although not statistically significant, results showed a trend that the freeze-dried spruce sprout powder contains more vitamin K_1_ compared to the fresh spruce sprouts (α = 0.05, *p* = 0.060 (in 2018) and *p* = 0.026 (in 2019)).

Vitamins and minerals of freshly frozen spruce sprouts were well preserved during the two-year storage period (–20 °C) (Table 3). In practice, these results indicate that entrepreneurs could have a stock for long-term storage of freshly frozen spruce sprouts and thus balance between the annual harvest, storage, and the quantity of final product manufacturing. However, the quality of the fresh raw material should be good and the storage conditions stable from the very beginning.

#### 2.6.4. Antioxidant Activity

**Figure 6:**
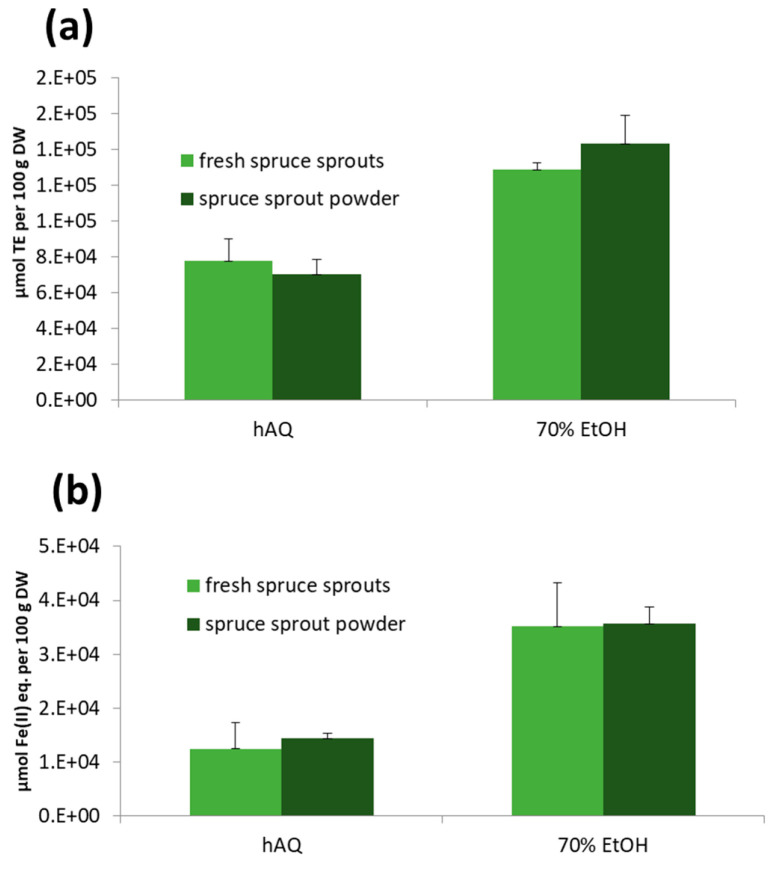
Oxygen radical absorbance capacity (ORAC) (**a**) and ferric ion reducing antioxidant power (FRAP) (**b**) of fresh sprouts and freeze-dried sprout powder of Norway spruce after water (hAQ) and 70% ethanol (70% EtOH) extractions in 2017–2019. The results are calculated per 100 g dry weight (DW). The error bars indicate standard deviations.

As with vitamins and minerals, a three-year antioxidative capacity control of the samples verified the stability of the quality of the spruce sprouts and their final product, the freeze-dried powder. As mentioned in Section 2.4., the highest ORAC and FRAP antioxidative capacities were found after ethanol-extractions (Figure 6). Otherwise, there were no significant antioxidative capacity differences between the fresh sprouts and the freeze-dried powder of Norway spruce (α = 0.05, *p*-values 0.6134 and 0.5981, respectively) in ORAC and FRAP tests after water (hAQ) or 70% ethanol extraction.

### 2.7. Characteristics of Food Product Prototypes with Sprouts and Needle Additions 

#### 2.7.1. Sensory Evaluation of Prototypes: Sprout and Needle Syrups and Ice-Creams 

Several syrup prototypes were made and evaluated in conjunction with their production at the Natural Resources Institute Finland (Luke) food product development and sensory laboratory (Jokioinen, Finland). Syrup prototypes varied by their spruce-based and commercial sucrose, glucose, and/or fructose ingredients and recipes. Two syrup prototypes were chosen for the ice-cream prototypes based on their flavor, consistency, and color attributes. 

Prototype ice-creams for sensory evaluation were made using typical dairy ice-cream base ingredients combined with spruce sprout or spruce needle syrups, or other conifer tree-based ingredients. The prototype products were evaluated and scored by trained sensory evaluation panelists (10 persons) at Luke, with reference ice-creams purchased from the local grocery store. Ice- cream portions were served to the panelists in blind-labeled cups and a randomized order. The ice-cream prototypes, reference products, and their evaluation data are compiled in Table 4.

The texture of the ice-creams made with syrup prototypes suffered from poor emulsion structure and/or poor aeration of the ice-cream mass, due to the substantial amount of the added syrup (10%). The poor texture is partly due to the low mixing capacity of the 2 liter-scale ice-cream machine. Most panelists remarked that the texture was grainy or watery, and the melting behavior was not typical for a dairy-based ice-cream. The panelists gave the following flavor attributes: caramel, cereal, and cooked, all describing typical properties of a syrup. None of the panelists mentioned typical flavors associated with the fresh or dried sprouts or conifer needles. Despite the sensory panel outcome, the scores of overall quality, expressed as “willingness to purchase” in Table 4, were for three prototypes similar to those of the commercial reference ice-creams.

#### 2.7.2. Ice-Creams with Fresh Sprouts Powder: Consumer Acceptance 

Based on the trained sensory panel results (above), Arctic Ice-cream Factory Oy, an SME company in Rovaniemi (Finland), made sprout ice-cream and sprout sorbet for the consumer acceptance survey by using the fresh sprouts. A third product for the consumer acceptance survey was an ice-cream with forest berries, made by the same company. 

A total number of 1197 respondents evaluated the products (Table 5). From the respondents, 63.8% were females and 36.2% males, and age groups (years) were: 0–6, 7–13, 14–17, 18–24, 25–40, 41–60, >60. Almost half of all respondents (48.1%) were children under 14. From them, 55.1% were female and 44.9% male. Another large group (23.7%) were adults between the age of 25 and 40 years, and 75% of them were female. 

**Figure 7:**
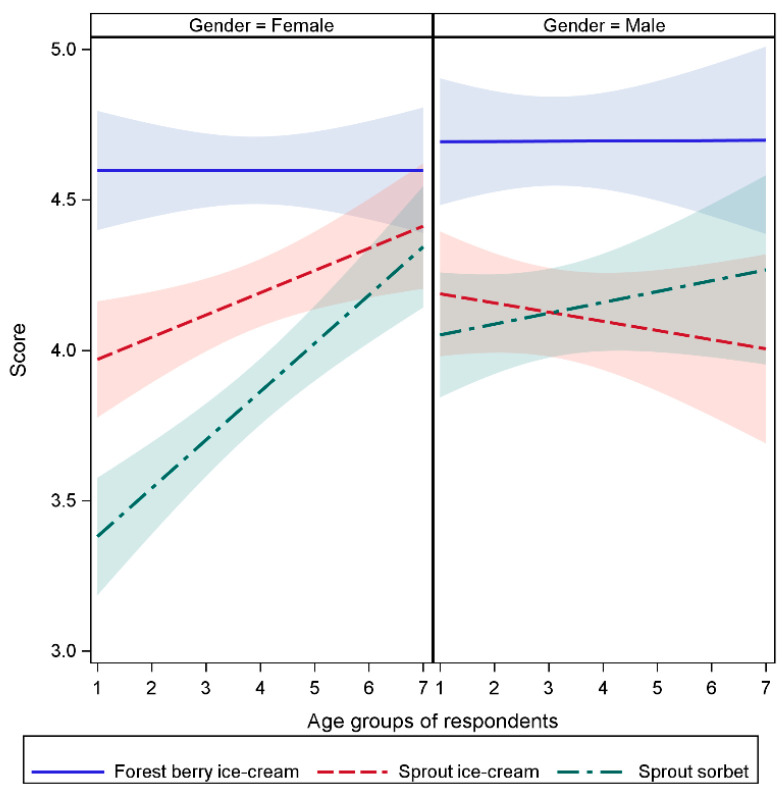
Differences of sensory evaluation scores between gender and age groups of respondents. The evaluated products were cream-based ice-creams with forest berries (blue lines) and sprouts (red lines), and sorbet with sprouts (green lines). The numbers on the x-axis refer to the age groups of the respondents (years): 1, 0–6; 2, 7–13; 3, 14–17; 4, 18–24; 5, 25–40; 6, 41–60; 7, >60.

The results from the survey showed that ice-creams with fresh sprout additions were described as very good or good (75% of females and 78% of males). Sorbet was slightly preferred over ice-cream with sprouts among male respondents (scores 4 and 5 by 78% and 77%, respectively; Table 5; Table 6, Figure 7). For female respondents, in contrast, the sprout ice-cream received slightly higher scores than sprout sorbet (scores 4 and 5 by 82% and 67%, respectively; Table 6). For females, the scores of sprout products increased with increasing age (Figure 7). For males, no clear age-dependency could be observed. The reference ice-cream with forest berries, however, received the highest scores in both gender groups and across all age groups (Table 5, Figure 7). This was an expected result, as this type of flavor is very popular in dairy-based yogurts, desserts, and ice-cream in Finland and other Nordic countries. 

## 3. Materials and Methods 

### 3.1. Chemicals

Unless otherwise stated, the chemicals were purchased from VWR International (Helsinki, Finland).

### 3.2. Plant Raw Materials 

**Figure 8:**
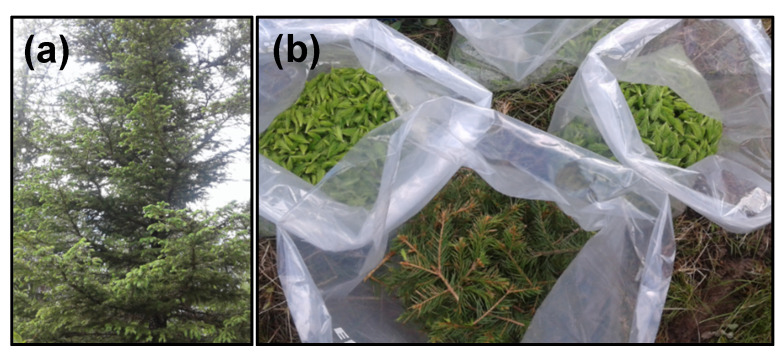
A typical young sample tree of Norway spruce in Nothern Finland (**a**): sprouts and needles were collected into plastics bags and immediately put into a freezer (**b**).

Norway spruce (*Picea abies* [L.] Karst.) sprout and needle samples were obtained from young trees grown in Northern Finland, Narkaus village in Rovaniemi (66°16.823′N, 26°6.818′E, 197 m above sea level (a.s.l); Figure 8). The sample collection was carried out in late-June (27) 2017. The quality controlling samples of the production-chain were harvested every year at the same time in late-June as part of the foodstuff production chain in 2017–2019.

**Figure 9:**
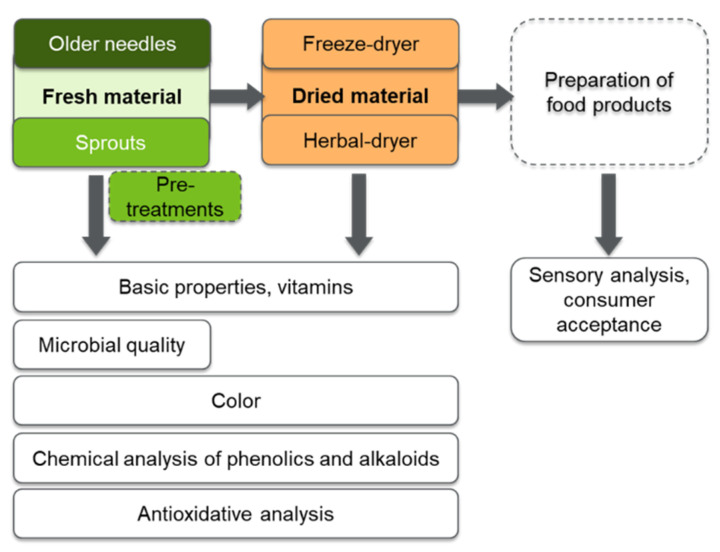
Preparation of the Norway spruce sprouts and needles for different analysis. Pre-treatments: manual removal of matter other than sprouts/needles, e.g., woody stem parts.

Sprouts and needles were manually harvested from several individual trees at the height of ca. 0.5–2.5 m on the stem, avoiding defects (i.e., insects, dried needles). Harvesting for research purposes was carried out along with the forest owner’s own harvest for commercial purposes, using the same protocols (Figure 8). The sprouts and needles were collected wearing clean gloves, and no mosquito repellents or other insecticides were used to verify purity of the collected raw materials. The raw materials of several collectors were mixed. After returning from the forest, the sprouts and needles were immediately placed at –18 °C overnight. On the following day, the samples were transported in closed containers protected from light at 2 to 4 °C and directed for further processing. One fourth of the raw material was immediately stored at –80 °C, one forth was directed to the analysis as fresh (basic properties: color, dietary fiber content, and microbial quality), and the rest of the sample mass was first dried (freeze-drying, herbal dryer-drying) and stored at –80 °C prior to further processing (Figure 9). The dry matter content was determined from fresh and differently dried samples. 

During the quality control of the whole production chain of Arctic Warriors (Arctic Warriors Oy, Narkaus, Lapland, Finland) in 2017–2019, the average amount of the harvested spruce sprouts was 3000–5000 kg and the harvesting period was around one week. The spruce sprouts were frozen (–20 °C) immediately after returning from the forest and were transported as a refrigerated batch to freeze-drying in Estonia. Freeze-drying process details are undisclosed due to the request of the freeze-drying company. The quality control samples were taken freshly from the forest and after the freeze-drying in an extensive manner. The quality control of the raw materials included safety factors (e.g., microbiological quality and traces of pesticides and heavy metals) and healthiness (e.g., macro-components, dietary fibers, vitamins, minerals, and antioxidant activity).

### 3.3. Drying Treatments

#### 3.3.1. Freeze-Drying

Freeze-drying of sprouts and needles was carried out at an industrial facility (Nature Lyotech Oy, Espoo, Finland; https://lyotech.com/naturedry.html) with NatureDry© freeze-drying technology by using frozen samples (Parker Freeze Dry, Inc., Pulaski, WI, USA). NatureDry© is a combination of laboratory freeze-drying methods and technology allowing freeze-drying of food products including berries, fruit, and vegetables without exposing them to high temperatures or excessive intracellular vapor pressure during the drying process. This allows preserving the authentic flavor and nutrients of the raw materials. The drying of the raw materials was carried out with a multi-dryer freeze-drier (Frozen in Time, York, North Yorkshire, Great Britain). First, the frozen samples were placed on trays and the temperate was stabilized at –25 °C. The primary drying was done at –25 °C for 4 days, followed by the secondary drying at 25 °C for ca. 1.5–2 days.

#### 3.3.2. Condensing Circulating Warm Air-Drying (Warm Air Dryer)

Another part of sprouts and needles was dried with a condensing circulating warm air-dryer (VegeDryer 100, Siikaisten Ykkösmyymälät Oy, Siikainen, Finland), intended for food and vegetable drying (referred to as herbal drier in the text). Isothermic drying at 35 °C took circa 46 h. At the end of the drying process, the relative humidity (RH) in the drying cabin was 23% (at 35 °C). The samples were cooled to 25 °C, and dry mass and color were determined. The samples were then packed into plastic bottles (high-density polyethylene (HDPE) with low-density polyethylene (LDPE) caps) and stored at –80 °C.

### 3.4. Microbial Quality

The microbial quality of fresh sprouts and older needles of Norway spruce were analyzed in Luke’s laboratory. The samples for microbiological analyses were mixed and 10 g was weighed by a Baby Gravimat gravimetric dilutor in stomacher bags and mixed with 90 mL of ¼-strength Ringer solution (Merck 1.15525.0001, Darmstadt, Germany). The samples were homogenized with stomacher (Stomacher^®^ 400 Circulator) for 2 min at 230 rpm. Serial decimal dilutions were prepared by mixing 1 mL of sample with 9 mL of Ringer solution. Enterobacteria were determined by the method described in International Organization for Standardization (ISO) 21528-2:2004 on Violet Red Bile Glucose Agar medium (Lab M Ltd., LAB088, Lancashire, UK). The petri dishes were incubated at 37 °C and the colonies were counted after 24 h. The typical colonies were counted, but not confirmed. The aerobic plate count was determined by the method described in ISO 4833-1:2013 on Plate Count Agar (Lab M Ltd., LAB010, Lancashire, UK) dishes incubated at 30 °C for 72 h. Yeasts and molds were determined by the method described in ISO 21527-1:2008 on Dichloran Rose Bengal Chloramphenicol Agar medium (DRBC Agar, LAB217, Lab M Ltd., Lancashire, UK), which was supplemented with 50 μg/mL of oxytetracycline hydrochloride (AppliChem BioChemica A5257, Darmstadt, Germany). The petri dishes were incubated at 25 °C. The colonies were counted after 3 and 5 days. 

The microbial quality control samples in 2017–2019 were analyzed by using standard methods at accredited laboratories (Eurofins Scientific Finland Oy, Helsinki, Finland). The following analyses were included: *Salmonella* bacteria, *Listeria monocytogenes* bacteria, Clostridium perfringens, *Escherichia coli* bacteria, and *Bacillus cereus* bacteria.

### 3.5. Basic Properties and Dietary Fiber Content

Basic properties and dietary fiber content were determined from both the fresh raw materials and the dried sprouts and needles. The basic properties were analyzed by using standard methods at accredited laboratories (Eurofins Scientific Finland Oy, Helsinki, Finland). The following analyses were included: macro-components (energy—calculated, fat—total, carbohydrate—available, and protein—total), carbohydrate components (glucose, galactose, fructose, saccharose, lactose, maltose), minerals (P, K, Ca, Cu, Mg, Na, Fe), and vitamin C. Dietary fiber (DF) content was analyzed at the Finnish Food Authority by a standard method according to the Association of Official Analytical Chemists (AOAC) official method 2011.25 [43,44]. Freeze-dried spruce sprouts and needles were milled through a 0.5 mm sieve. Starch and protein were removed by incubating the samples in a shaking water-bath (α-amylase/amyloglucosidase (AMG), for 16 h at 37 °C, and protease, for 30 min at 60 °C; Megazyme, Bray, Ireland). The enzymatically treated samples were first filtered (fritted crucibles with coarse (American Society for Testing and Materials (ASTM)) 40–60 µm pore size) to separate water-insoluble DF (IDF) from water-soluble DF (SDF), and a second time to separate water-soluble polysaccharides (SDFP) from oligosaccharides (SDFS). IDF and SDFP were dried, weighed, and corrected with protein and ash values. SDFS was further hydrolyzed using AMG, deionized, and analyzed by high-performance liquid chromatography (HPLC), as described in Rainakari et al. [45]. The total DF amount is the sum of IDF, SDFP, and SDFS.

In addition, the quality control samples in 2017–2019 were analyzed by using standard methods at accredited laboratories (Eurofins Scientific Finland Oy). The following analyses were included: vitamins (C, K1, and beta carotene, i.e., vitamin A precursors total, cis + trans) and minerals (Zn, Fe, Mg, Ca, K, P). 

### 3.6. Pesticides and Heavy Metals

The quality control samples in 2017–2019 were analyzed by using standard methods at accredited laboratories (Eurofins Scientific Finland Oy, Helsinki, Finlad). The following analyses were included: pesticides (e.g., pyrethroids and organochloride, nitrate, and phosphorus compounds) and heavy metals (As, Hg, Cd, and Pb).

### 3.7. Determination of Color

Fresh and dried sprouts and needles were analyzed for their color parameters in the visible light wavelength range 400–700 nm by a Minolta CM-508s spectrophotometer equipped with a SpectraMagic software (Konica Minolta, Tokyo, Japan), which is using a CieLab method (International Commission on Illumination L*a*b* scale). In this method, the spectrophotometer is measuring the samples from the top of the plate samples where the samples are spread, and the software calculates values for sample color lightness (L*, scale 100...0) and actual color in red-green (a*, scale +100…–100) or yellow-blue (b*, scale +100…–100) color spaces. From these values, the intensity of the color (chroma, C*) and its shade (hue angle, h) were calculated. A graph illustrating L*, a*, b*, and C* was chosen best to describe the differences of sprout and needle samples (Figure 2). Each sample was measured in 5 replicates.

### 3.8. Analysis of Antioxidative Properties of Spruce Sprouts and Needles

Antioxidant properties of freshly frozen, freeze-dried, and sprouts and needles dried in an herbal dryer were assessed by indirect methods to screen different antioxidant mechanisms. Indirect methods included a single electron transfer-based (SET), a hydrogen atom transfer-based (HAT) (FRAP and ORAC), and hydrogen peroxide scavenging assays (SCAV).

Samples were first powdered by grounding them in liquid nitrogen before extraction. The extract solution of 70% ethanol (aqueous EtOH) or hot water (hAq) was pipetted onto the powder in a test tube (1:10), vortexed for 2 min, incubated for 15 min at room temperature (RT), and again vortexed for at least 2 min, after which the tubes were centrifugated at 8400 rpm for 10 min (Sigma 2-16KL). The surface solution was pipetted into a different tube, and the sample was collected from the surface solution.

#### 3.8.1. FRAP

Differences in the antioxidant activity between the freshly frozen and differently dried sprout and needle extracts were measured using a single electron transfer-based FRAP (ferric ion reducing antioxidant power) method, which measures the ability of an antioxidant to reduce ferric (FeIII) to ferrous (FeII) ions [46]. The reaction mixture contained the sample, 20 mmol/L FeCl_3_·6H_2_O (Sigma-Aldrich Chemie GmbH, Steinheim, Germany) and 10 mmol/L 2,4,6-Tris (2-pyridyl)-s-triazine (TPTZ) (Sigma-Aldrich Chemie GmbH, Steinheim, Germany) in 300 mmol/L acetate buffer pH 3.6. The formation of ferrous-tripyridyltriazine complex in the reaction mixture is measured by absorbance at 593 nm in 96-microplate format with three technical replicates of each sample on the plate and series of dilutions to fit the sample to the standard curve. FeSO_4_·7H2O (Sigma-Aldrich Chemie GmbH, Steinheim, Germany) was used as a standard compound and L(+)-ascorbic acid (150 µM and 800 µM) (VWR Chemicals) as a control, and the results are expressed as µmol/L Fe(II) equivalents.

#### 3.8.2. ORAC

The oxygen radical absorbance capacity (ORAC) assay is a hydrogen atom transfer-based method, which measures the oxidative dissociation of fluorescein at the presence of peroxyl radicals (R-O-O•), which causes a reduction in the fluorescence signal. The antioxidant’s protective ability is based on the inhibition of the breakdown of fluorescein caused by the peroxyl radicals. The assay was modified from the method described by Huang et al. [47] and Prior et al. [48] and carried out in 96-well format with two technical replicates of each sample on the plate. Each reaction mixture contained 25 µL of the sample in 0.075 M phosphate buffer pH 7.5 (Merck), 150 µL of 8.16 × 10^–5^ mM fluorescein (Sigma-Aldrich Chemie GmbH, Steinheim, Germany), and 25 µL of 2,2′-Azobis(2-methylpropionamidine) dihydrochloride (Sigma-Aldrich Chemie GmbH, Steinheim, Germany). For each sample, a protocol with a series of five dilutions (from 1:1 to 1:320) was used and additional dilutions if needed to adjust the sample concentration to the standard curve. 0.153 mM Trolox ((±)-6-Hydroxy-2,5,7,8-tetramethylchromane-2-carboxylic acid, vitamin E analog) (Sigma-Aldrich Chemie GmbH, Steinheim, Germany) was used as a standard compound and the results are expressed as Trolox equivalents (µmol/L TE). Vitamin C (L(+)-ascorbic acid; Merck KGaA, Darmstadt, Germany) was used as a reference compound.

#### 3.8.3. H_2_O_2_ Scavenging

The hydrogen peroxide (H_2_O_2_) scavenging activity, based on transition metal chelation, was determined by using a method modified from Hazra et al. [49] and Jiang et al. [50] with a microplate reader in 96-well format with four technical replicates on each plate. An aliquot of 2 mmol/L H_2_O_2_ (Merck KGaA, Darmstadt, Germany) was added to the reaction mixture with the sample, 2.56 mmol/L ammonium iron (II) sulphate·6H_2_O (BDH Prolabo) and 111 µM xylenol orange disodium salt (Sigma-Aldrich Chemie GmbH, Steinheim, Germany). After 30 min incubation, the absorbance of the ferric-xylenol orange complex at 560 nm was measured. The assay measures the ability of the sample to scavenge H_2_O_2_ and prevent the oxidation of Fe(II) to Fe(III), which is indicated by the formation of the ferric-xylenol orange complex. The H_2_O_2_ scavenging ability is expressed as inhibition percentage (%) of Fe(II) oxidation to Fe(III). Sodium pyruvate (Sigma-Aldrich Chemie GmbH, Steinheim, Germany) was used as a reference compound.

### 3.9. Preparation of Food Prototype Products with Sprout and Needle Additions

For the preliminary sensory analysis, we prepared needle syrups by using the raw materials (a) freeze-dried sprouts, (b) sprouts dried using a condensing warm air-dryer, (c) freeze-dried needles, and (d) needles dried using condensing warm air-dryer (Table 7). First, a half of a liter of fresh spruce needles was collected and chopped into small pieces by using a stick mixer, and dry matter content was determined. Based on the results, a syrup recipe was applied (i.e., recipe for 1 L of fresh needles). The required dry mass of the dried raw materials was weighed, chopped with the stick mixer, and 1.5 L of cold water was added. The mixture was cooked at a mild temperature under a lid for circa 15 min until the color faded/turned brown. The liquid was then filtered, and sugar and glucose syrup were added (Table 7), and the mixture was cooked into a thick syrup, occasionally stirring (without lid, electric hot plate with medium heat). The mixture thickened into a syrup after approximately two hours; in the final step, the viscosity was tested by cooling a small amount of the mixture, as the final viscosity can only be determined from the product at room temperature.

Ice-cream prototypes included typical ingredients used in the manufacture of dairy-based high-quality ice-cream. A 2 L ice-cream machine (small pilot) and recipes from Arctic Ice-Cream Factory Oy were used. Prototypes were made with two spruce sprout/needle syrups, and the syrups were added into the ice-cream base before mixing. Additional ice-cream prototypes were also made using other conifer-based ingredients: extracts and ground powders of pine or spruce bark. “Pettu”, a traditional natural product in Nordic countries, consists of soaked, dried/warm smoked, and ground inner bark of Scots pine (*Pinus sylvestris* L.). Some of these ingredients were added to the ice-cream base, and some of them were mixed into the ice-cream as a spice before packaging. All prototype variants and the amount of added conifer ingredients are listed in Table 4, Section 2.7. 

For the final sensory analysis and consumer acceptance survey, fresh sprouts were used for preparing cream-based gourmet ice-cream and water-based sorbet with the following recipes: (a) in ice-cream, 15 g/kg sprouts were added to a gourmet ice-cream (to base or to ice-cream), (b) for sorbet, 8 g/kg sprouts were added to base, and (c) × g/kg forest-berries were added to a gourmet ice-cream. All recipes were developed, and products for consumer acceptance tests were made by Arctic Ice-Cream Factory Oy.

### 3.10. Sensory Analysis and Consumer Acceptance of Products

The outlook, odor, taste, and texture of the sprout and needle syrups were evaluated by a panel at the Luke sensory evaluation laboratory (Jokioinen, Finland), consisting of people involved in food product development activities on a weekly basis. The laboratory meets the ISO 8589:2007 standard (ISO 8589:2007 Sensory analysis—General guidance for the design of test rooms). 

Ice-cream prototypes were evaluated by trained sensory evaluation panelists at the same laboratory, using ice-creams purchased from the local grocery store as a reference. Details can be found in Table 4. Panelists evaluated the products individually, but in the same order, using non-structured descriptions (free wording). Each product was evaluated by its textural characteristics including outlook/outer surface structure, consistency/ability to cut by a spoon, and mouthfeel (e.g., melting, thickness, creaminess, graininess) using verbal descriptions. Aroma/flavor/taste was also verbally described. In addition to the verbal description, which each panelist individually filled in on the evaluation form, a score from 1 to 5 was given for each attribute, where 5 is the highest score. Liking of the products was expressed by willingness to purchase, using a YES/NO scale. The number of panelists who answered YES is included in the Table 4, where the average values of the scores are also shown. After evaluation of all products, comments were discussed using a round table format, and scores and comments were collected to the common commenting table. The main comments can be found in the results in Section 2.7. 

The consumer acceptance survey was carried out in an outdoor event “Mansikki” (Children’s agricultural exhibition) co-organized by Luke in early September 2019. The following three products were included in the randomized evaluation: (a) creamy ice-cream with sprout addition, (b) sorbet with sprout addition, and (c) a forest berry ice-cream (used as a reference). Scoring was done from 1 to 5. Evaluation points were classified as follows: 5 = very good, 4 = good, 3 = not good/not bad, 2 = bad, and 1 = very bad.

Consumers filled in the evaluation form individually, or, with the help of a parent/grandparent. Data was collated, statistically analyzed, and is shown in Table 5; Table 6, and Figure 8.

### 3.11. Statistical Analysis 

Generalized linear models (GLM) were used to evaluate whether there was evidence that the means of the material (i.e., sprouts vs. older needles), drying method (freshly frozen, freeze-dryer, herbal dryer), and solvent (i.e., aqueous vs. 70% ethanol) differed for FRAP or ORAC. All main effects and two-way interactions were included in the models, but the interaction of method and solvent was omitted from both models based on an F-test (*p* > 0.40). A three-way interaction was not possible to test because of sample size. The assumption of gamma distribution (with a log link) was used due to skewed dependent variables. Multivariate Analysis of Variance (MANOVA) was not used because the variances of dependent variables differed, and that could not be effectively taken into account in the combined model. 

Simplified GLM with the same assumptions was used for vitamin analysis. The effects of powder vs. fresh sample were compared for four dependent variables (vitamins C and K, beta-carotenoid, antioxidative activities, ORAC, and FRAP). Because of only three replicates per treatment, non-parametric analysis of variance (ANOVA) was tested also, but differences of the applied methods were minor.

A linear model was also used for the analysis of ice-cream products having product (forest berry, sprout ice-cream, and sprout sorbet), gender (female vs. male), and age group (1 to 7) as continuous variables, and all their interactions as fixed effects. A parametric test was used, even though the rating scale was an ordinal 5-point Likert scale. Although our model was based on the assumptions of the normal distribution of ratings, the risk of violation of this assumption was reduced, notably because of the large sample size (N = 1197) [51].

All models were fitted by using the maximum likelihood estimation method. The method of Westfall [52] was used in multiple comparisons with a significance level of α = 0.05. The residuals were checked for normality using graphical figures, such as a boxplot. The analyses were performed using the GLIMMIX and NPAR1WAY procedure specified by the SAS Enterprise Guide 7.1 (SAS Institute Inc., Cary, NC, USA).

## 4. Conclusions

We produced a new knowledgebase for improving the raw-material quality and nutritive composition of spruce sprouts and needles in the value-chain from harvesting to processing and storage, and customer acceptance of dessert product prototypes. The results showed that with appropriately done drying of the raw material, the antioxidative properties of sprouts and needles can be well preserved. This will add value to both the food industry and the customers. Older needles generally showed a higher antioxidant activity, but lower vitamin and mineral content than the sprouts. The taste of light-green ‘Nordic gold’ sprouts is unique and can serve in culinary gourmet products. In the future, though, also older needles may be increasingly used for certain products, e.g., in industrial production of beverages, where the extraction of the raw material can be applied. More research and development are needed to enhance the usage and improve the quality control in the value-chain of both sprouts and needles. Also, phenolic analyses of the studied spruce sprouts and needles remain as a topic for further study.

## Figures and Tables

**Table 1:** Dry matter content of Norway spruce sprouts and older needles as fresh and after different drying treatments.

Drying Method	Raw Material	Dry Matter %
none, frozen	sprouts	16.58
freeze-drying	sprouts	95.35
warm air dryer	sprouts	93.18
none, frozen	needles	54.75
freeze-drying	needles	96.43
warm air dryer	needles	84.49

**Table 2:** Microbial quality of freshly collected Norway spruce sprouts and older needles. Colony counts log_10_ (colony forming units (cfu)/g) fresh weight (N = 1).

Raw Material	Enterobacteria	Aerobic Plate Counts	Yeasts	Molds
sprouts	<10	2.4	3.6	3.0
needles	<10	4.2	4.4	4.3

**Table 3:** Stability of vitamins and minerals of spruce sprouts (x ± standard deviation (SD)) during two-year storage at 20 °C.

Vitamin/Mineral	A. Freshly Frozen Spruce Sprouts Analyzed in 2018	B. Batch 2018 Analyzed in 2020 (Stored at –20 °C)	*p*-Values of Statistical Comparison of A and B (*α* = 0.05)
Vitamin K_1_ (μg/100 g DW)	140 ± 30	65 ± 13	0.146
Vitamin C (mg/100 g DW)	350 ± 40	290 ± 30	0.517
Beta carotene, vitamin A precursors total (cis + trans) (μg/100 g DW)	3700 ± 1100	4000 ± 1000	0.343
Zinc (mg/kg DW)	38 ± 8	37 ± 7	0.945
Iron (mg/kg DW)	23 ± 5	20 ± 4	0.756
Magnesium (mg/kg DW)	1100 ± 220	1300 ± 300	0.211
Calsium (mg/kg DW)	1640 ± 330	1300 ± 300	0.103
Potassium (mg/kg DW)	10,000 ± 2000	12,000 ± 3000	0.027
Phosphorus (mg/kg DW)	2600 ± 600	3000 ± 600	0.174

**Table 4:** Sensory evaluation and overall liking of prototype ice-creams and commercial ice-creams (used as a reference). Scores of 10 evaluators (x ± SD), evaluation scale 1–5 ^a^.

Ice-cream Prototypes (Samples)		Texture Characteristics		Odor, Taste	Willingness to Purchase
	Outlook	Hardness, spoonable	Mouthfeel		
Pine bark flour, 2 g/kg	3.4 ± 1.0	3.5 ± 0.8	3.1 ± 0.9	3.6 ± 0.9	2/10
“Pettu” pine bark as flavor, 2 g/kg	3.9 ± 0.9	3.7 ± 1.1	3.7 ± 0.7	3.9 ± 0.8	9/10
Spruce syrup prototype 2 as ingredient, 100 g/kg	3.9 ± 0.7	3.6 ± 0.7	4.1 ± 0.8	3.6 ± 0.9	6/10
Spruce syrup prototype 3 as ingredient, 100 g/kg	3.2 ± 0.8	3.2 ± 1.1	3.2 ± 1.1	3.6 ± 0.9	3/10
Spruce inner bark as flavor, 2 g/kg	3.1 ± 1.1	3.1 ± 0.9	3.5 ± 0.9	3.6 ± 1.0	3/10
Spruce inner bark as ingredient, 2 g/kg	3.8 ± 0.9	3.9 ± 0.7	4.0 ± 0.6	3.7 ± 1.1	5/10
**Commercial Ice-creams (Reference Samples)**					
Pingviini vanilla, 1 L brick	4.7 ± 0.5	4.2 ± 0.7	4.5 ± 0.5	4.5 ± 0.5	10/10
JYMY 1917, vanilla, organic, tub	4.6 ± 0.5	4.6 ± 0.5	4.7 ± 0.5	4.5 ± 0.9	9/10
JYMY 1917, pine extract flavor, tub	4.3 ± 0.7	4.5 ± 0.7	4.6 ± 0.7	3.6 ± 1.3	5/10

^a^ Sensory evaluation scale: 5, very good; 4, good; 3, acceptable; 2, bad; 1, very bad.

**Table 5:** Results of consumer acceptance evaluation of frozen desserts with sprouts (ice-cream and sorbet) and reference product (forest berry ice-cream). The sum of respondents’ score ^a^ (1—5). ^a^ Sensory evaluation scale: 5, very good; 4, good; 3, acceptable; 2, bad; 1, very bad.

	Sprout Ice-Cream					Forest Berry Ice-Cream					Sprout Sorbet					
**Female**	**Score**					**Score**					**Score**					**TOTAL**
**Age years**	**1**	**2**	**3**	**4**	**5**	**1**	**2**	**3**	**4**	**5**	**1**	**2**	**3**	**4**	**5**	
0–6	1	4	3	4	22		2	1	4	22	1	5	7	6	13	95
7–13	3	5	14	28	25	1	1	5	12	57	9	10	20	21	15	226
14–17			1	2				2		1			1	2		9
18–24			1	3	4				4	6			2	3	4	27
25–40		2	6	34	28			2	21	48	1	3	19	25	24	213
41–60			3	18	21			2	15	25		2	2	19	28	135
>60			3	7	10			2	4	14		1	2	11	5	59
**total**	**4**	**11**	**31**	**96**	**110**	**1**	**3**	**14**	**60**	**173**	**11**	**21**	**53**	**87**	**89**	**764**
**Male**	**Score**					**Score**					**Score**					
**Age years**	**1**	**2**	**3**	**4**	**5**	**1**	**2**	**3**	**4**	**5**	**1**	**2**	**3**	**4**	**5**	**45**
0–6	1	1	5	14	22	1		4	3	33	3	5	1	11	23	127
7–13	1	2	6	16	20			2	8	35	1	3	8	13	20	135
14–17		2	1	1					1	2	1			2		10
18–24			2	1						3				1	2	9
25–40		1	5	7	10				4	21		1	2	8	12	71
41–60			3	7	6			1	7	8		1	2	4	9	48
>60			3	5	3				3	8			4	3	4	33
**total**	**2**	**6**	**25**	**51**	**61**	**1**	**0**	**7**	**26**	**110**	**5**	**10**	**17**	**42**	**70**	**433**

**Table 6:** Statistical analysis of consumer acceptance evaluation results of frozen desserts with sprouts (sprouts ice-cream and sorbet) and a reference product (forest berry ice-cream).

Observed Result	*p*-Value
‘Forest berry’ scores did not differ by gender	0.6498
‘Sprout’ scores did not differ by gender	0.7944
‘Sorbet’ scores differed by gender	0.0013
Women liked ‘forest berry’ the most	<0.0001
Women liked ‘sprout ice-cream’ more than ‘sorbet’	<0.0001
Men liked ‘forest berry’ the most	<0.0001

**Table 7:** Recipe for syrups of differently dried Norway spruce sprouts and needles.

Drying Method	Raw Material	Dry Matter %	Amount for 1.5 L (g)	Added Sugar Types	Amount (g)
freeze-drying	sprouts	95.35	75	saccharose	450
warm air-dryer	sprouts	93.18	78	saccharose, glucose syrup	330 441
freeze-drying	needles	96.43	75	saccharose	450
warm air-dryer	needles	84.49	84	saccharose, glucose syrup	330 441
